# Supplementary material for: Intracellular accumulation of amyloid-ß is a marker of selective neuronal vulnerability in Alzheimer’s disease
Source: Nat Commun. 2025 Jun 4;16:5189. doi: 10.1038/s41467-025-60328-w (PMC12137956; doi:10.1038/s41467-025-60328-w)
Supplement: Supplementary file 1 — Supplementary Information [file 41467_2025_60328_MOESM1_ESM.pdf]

Supplementary Figures

Intracellular accumulation of amyloid-ß is a marker of selective neuronal vulnerability in Alzheimer's disease

Alessia Caramello[1,2,3], Nurun Fancy[1], Clotilde Tournerie[1], Maxine Eklund[1], Vicky Chau[1], Emily Adair[1], Marianna Papageorgopoulou[1], Nanet Willumsen[1], Johanna S. Jackson[1], John Hardy[2], Paul M. Matthews[1,4*]

[1] UK Dementia Research Institute Centre at Imperial College London and Department of Brain Sciences, 728 Sir Michael Uren Research Hub, 86 Wood Ln, London W12 0BZ, UK
[2] UK Dementia Research Institute Centre at University College London, Department of Neurodegenerative Disease, Wing 1.2 Cruciform Building, Gower Street, London, WC1E 6BT, UK
[3] Laboratory of Stem Cell Biology and Developmental Genetics, The Francis Crick Institute, 1 Midland Road, London, NW1 1AT, UK
[4] The Rosalind Franklin Institute, Harwell Science and Innovation Campus, Fermi Way, Didcot, Oxon. OX11 0QS, UK

*** Corresponding author:**
Prof Paul M. Matthews
E515, Department of Brain Sciences
Imperial College London
Hammersmith Hospital
DuCane Road, London WC12 0NN
UK
Tel: 0044 207 594 2612
p.matthews@imperial.ac.uk

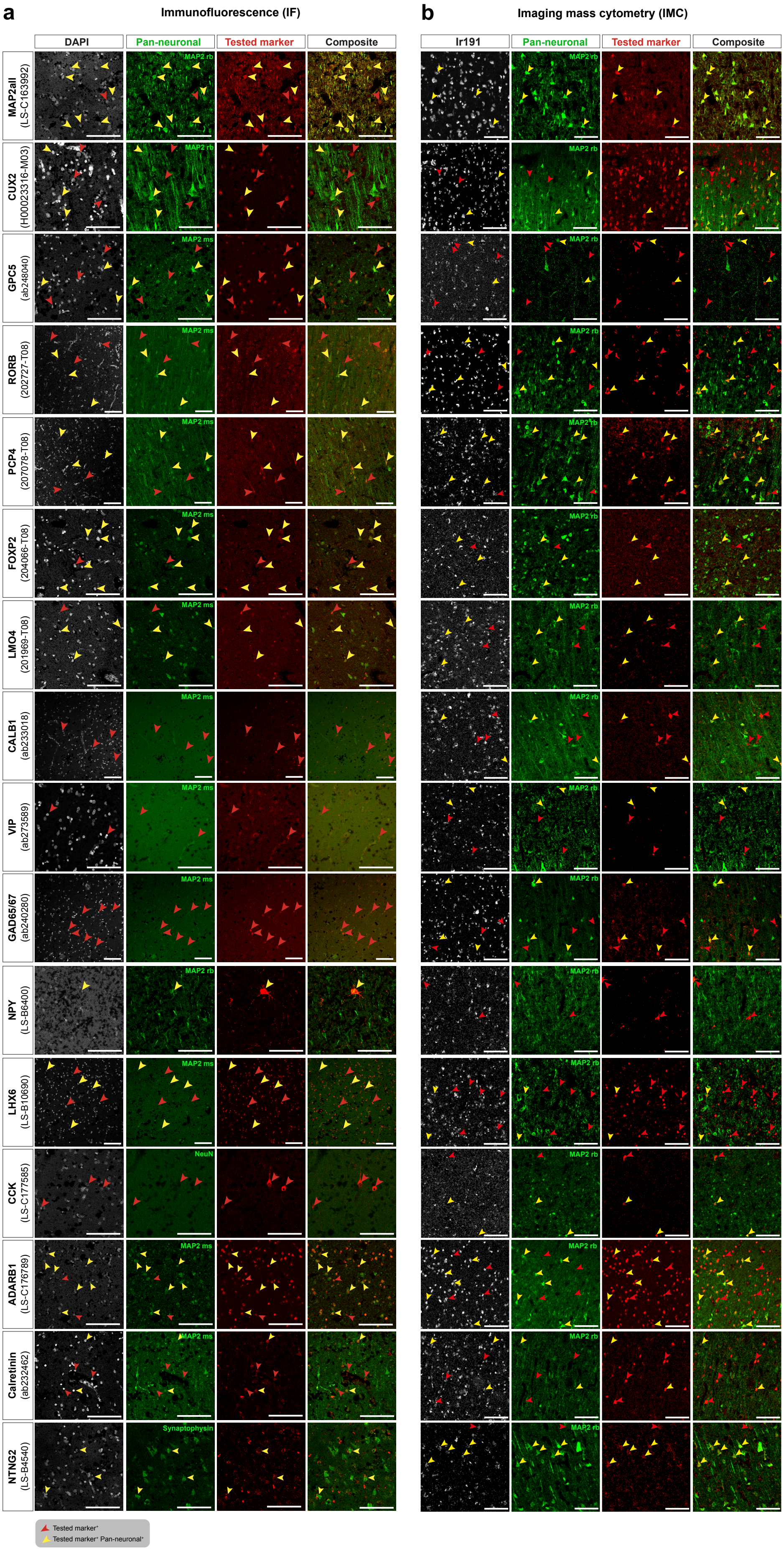
a
Immunofluorescence (IF)
b
Imaging mass cytometry (IMC)
DAPI
Pan-neuronal
Tested marker
Composite
Ir191
Pan-neuronal
Tested marker
Composite
MAP2all (LS-C163992)
CUX2 (H00023316-M03)
GPC5 (ab248040)
RORB (202727-T08)
PCP4 (207078-T08)
FOXP2 (204066-T08)
LMO4 (201969-T08)
CALB1 (ab233018)
VIP (ab273589)
GAD65/67 (ab240280)
NPY (LS-B6400)
LHX6 (LS-B10690)
CCK (LS-C177585)
ADARB1 (LS-C176789)
Calretinin (ab232462)
NTNG2 (LS-B4540)
MAP2 rb
MAP2 ms
NeuN
Synaptophysin
Tested marker+
Tested marker+ Pan-neuronal+

Supplementary Figure 1. **Immunofluorescence (IF) and imaging mass cytometry (IMC) signal from 16 neuronal markers.** The shortlisted new neuronal markers (Fig.1b; Supp. Table 2) were tested by comparing their immunostaining in paired sections from the same brains with IF or IMC in conjunction with established pan-neuronal or synaptic markers (MAP2, MAP2all, NeuN or synaptophysin) and an iridium nuclear marker (Ir191). Only neuronal markers that were validated in this way are shown here (16/34 tested). These were used in the IMC panel applied for the studies described. The IMC antibodies used in these tests were conjugated to the same metal isotopes as the finalised panel (Supp. Table 3). Yellow arrowheads indicate cells double positive for the tested and pan-neuronal marker, while red arrowheads indicate cells only positive for the tested neuronal marker. Scale bars represent 100μm. Original figure and individually cropped images used to generate this figure are available on Figshare (https://doi.org/10.6084/m9.figshare.28955909.v2) and the full IMC images from which the cropped images were generated also is available on Figshare (https://doi.org/10.6084/m9.figshare.27909663.v1).

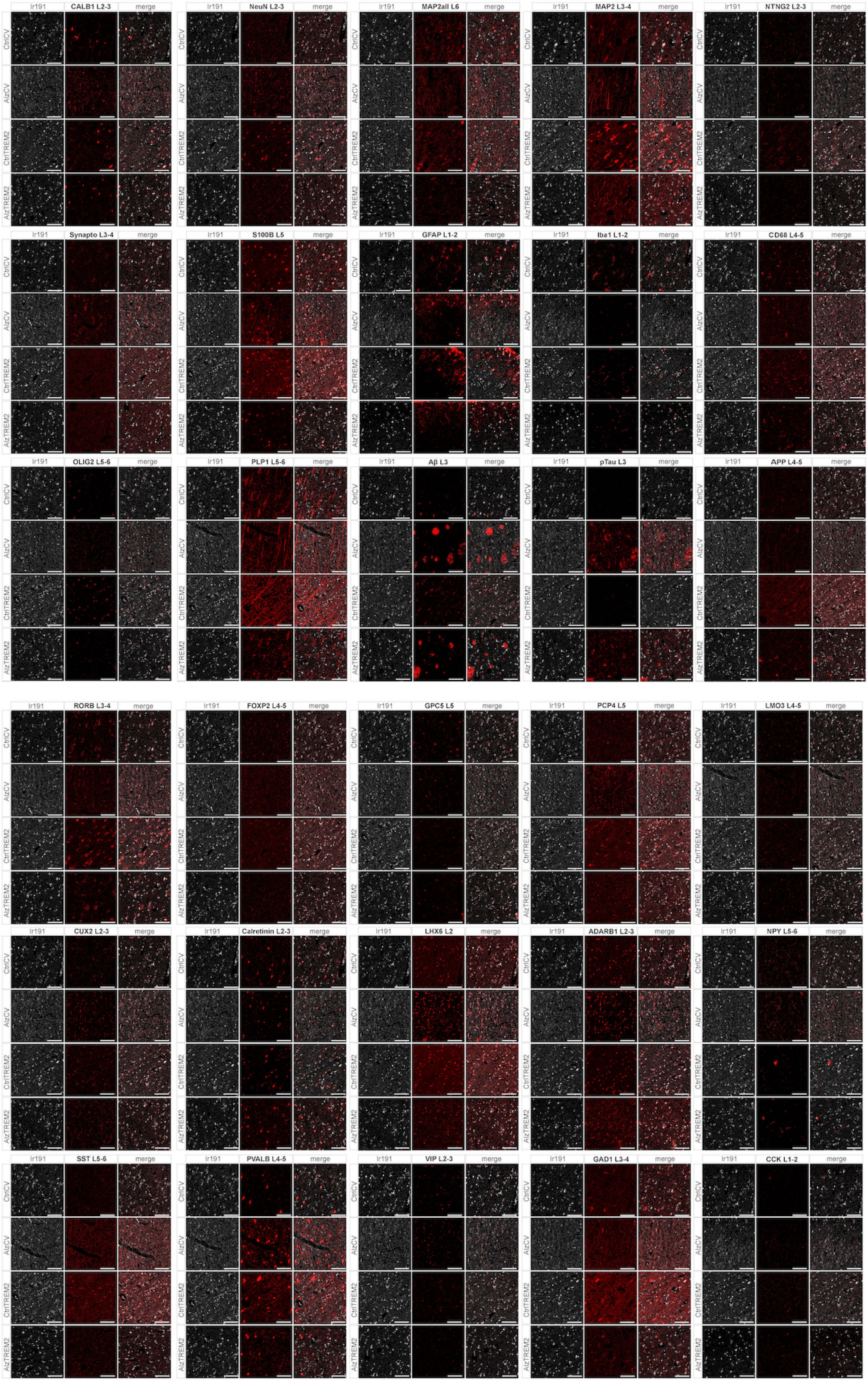
Ir191
merge
CtrlCV
AlzCV
CtrlTREM2
AlzTREM2
CALB1 L2-3
NeuN L2-3
MAP2all L6
MAP2 L3-4
NTNG2 L2-3
Synapto L3-4
S100B L5
GFAP L1-2
Iba1 L1-2
CD68 L4-5
OLIG2 L5-6
PLP1 L5-6
Aβ L3
pTau L3
APP L4-5
RORB L3-4
FOXP2 L4-5
GPC5 L5
PCP4 L5
LMO3 L4-5
CUX2 L2-3
Calretinin L2-3
LHX6 L2
ADARB1 L2-3
NPY L5-6
SST L5-6
PVALB L4-5
VIP L2-3
GAD1 L3-4
CCK L1-2

Supplementary Figure 2. **Multiplexed immunostaining IMC images.** Representative expanded sections of imaging mass cytometry (IMC) regions of interests from CtrlCV, CtrlTREM2, AlzCV and AlzTREM2 samples showing staining from all 31 antibodies in the panel used for these studies together with an iridium nuclear marker (Ir191). Scale bars represent 100μm. Original figure and individually cropped images used to generate this figure are available on Figshare (https://doi.org/10.6084/m9.figshare.28955909.v2) and the full IMC images from which the cropped images were generated also is available on Figshare (https://doi.org/10.6084/m9.figshare.27909663.v1).

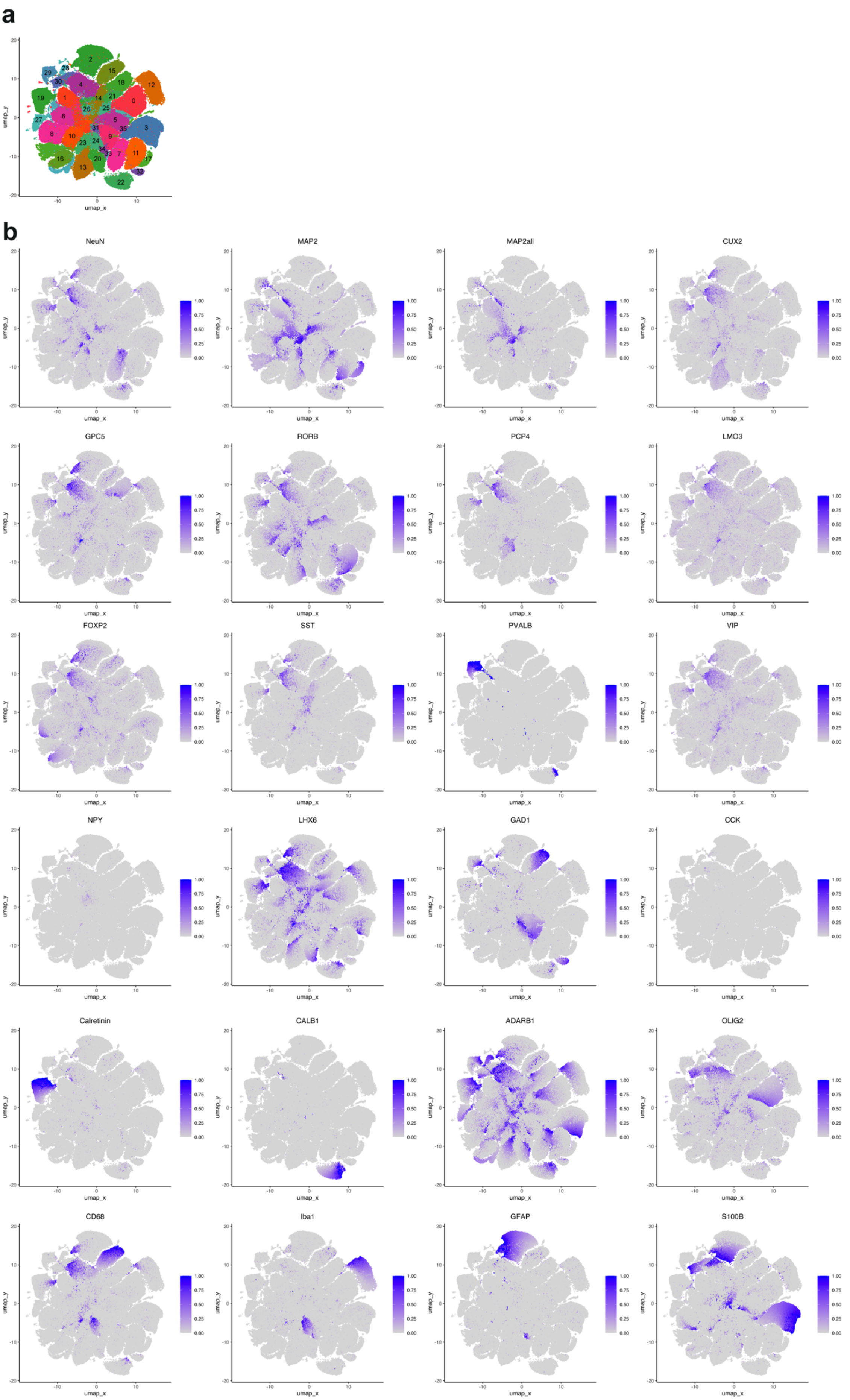

a
umap_x
umap_y
b
NeuN
MAP2
MAP2all
CUX2
GPC5
RORB
PCP4
LMO3
FOXP2
SST
PVALB
VIP
NPY
LHX6
GAD1
CCK
Calretinin
CALB1
ADARB1
OLIG2
CD68
Iba1
GFAP
S100B

Supplementary Figure 3. **Immunostaining marker expression across neuronal clusters. a** UMAP plot of the 35 clusters identified from all cells of all samples detected by imaging mass cytometry (IMC). **b** UMAP plots of neuronal and glial marker expression with clustered nuclei. Source data are provided as a Source Data file.

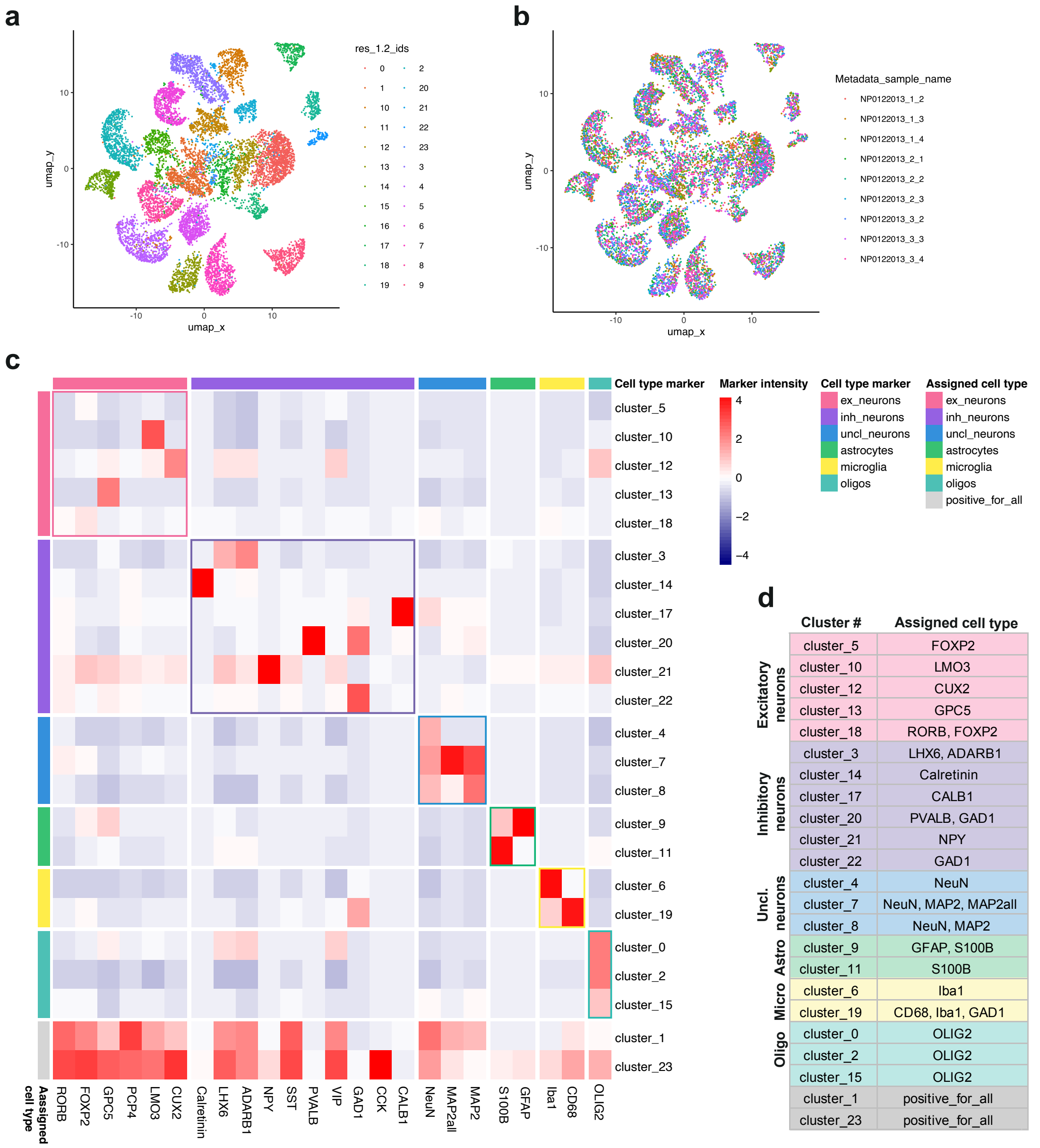

	Cluster #	Assigned cell type
Excitatory neurons	cluster_5	FOXP2
	cluster_10	LMO3
	cluster_12	CUX2
	cluster_13	GPC5
	cluster_18	RORB, FOXP2
Inhibitory neurons	cluster_3	LHX6, ADARB1
	cluster_14	Calretinin
	cluster_17	CALB1
	cluster_20	PVALB, GAD1
	cluster_21	NPY
	cluster_22	GAD1
Uncl. neurons	cluster_4	NeuN
	cluster_7	NeuN, MAP2, MAP2all
	cluster_8	NeuN, MAP2
Astro	cluster_9	GFAP, S100B
	cluster_11	S100B
Micro	cluster_6	Iba1
	cluster_19	CD68, Iba1, GAD1
Oligo	cluster_0	OLIG2
	cluster_2	OLIG2
	cluster_15	OLIG2
	cluster_1	positive_for_all
	cluster_23	positive_for_all

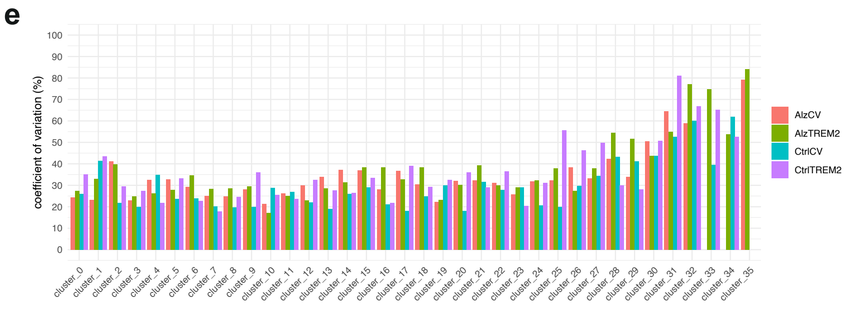

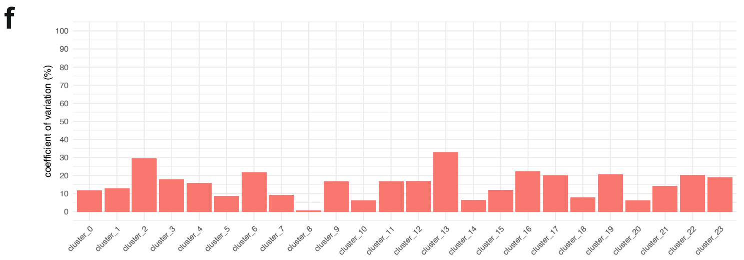

Supplementary Figure 4. **Testing accuracy and precision of our IMC antibody panel on 3 ROIs from 3 slides of a CtrlTREM2 sample. a,b** UMAP plots of clusters identified in this analysis, shown by cluster identity (a) and slide/region of interest (ROI) origin (b) (NP0122013_1 ROIs #2,3,4, NP0122013_2 ROIs #1,2,3, NP0122013_3 ROIs #2,3,4). **c** Heatmap of intensity of imaging mass cytometry (IMC) markers expression in each cluster and preliminary assignment to cell types. **d** Final assignment of clusters to neuronal and glial populations based on markers expression shown in (c). **e** Coefficient of variation (CV) of cell number per cluster between ROIs of the same individual from our original study dataset, grouped by disease (Ctrl/Alz) and TREM2 variants (CV/TREM2). **f** CV of cell number per cluster between slides from the same individual (CtrlTREM2 – NP0122013_1, NP0122013_2, NP0122013_3).

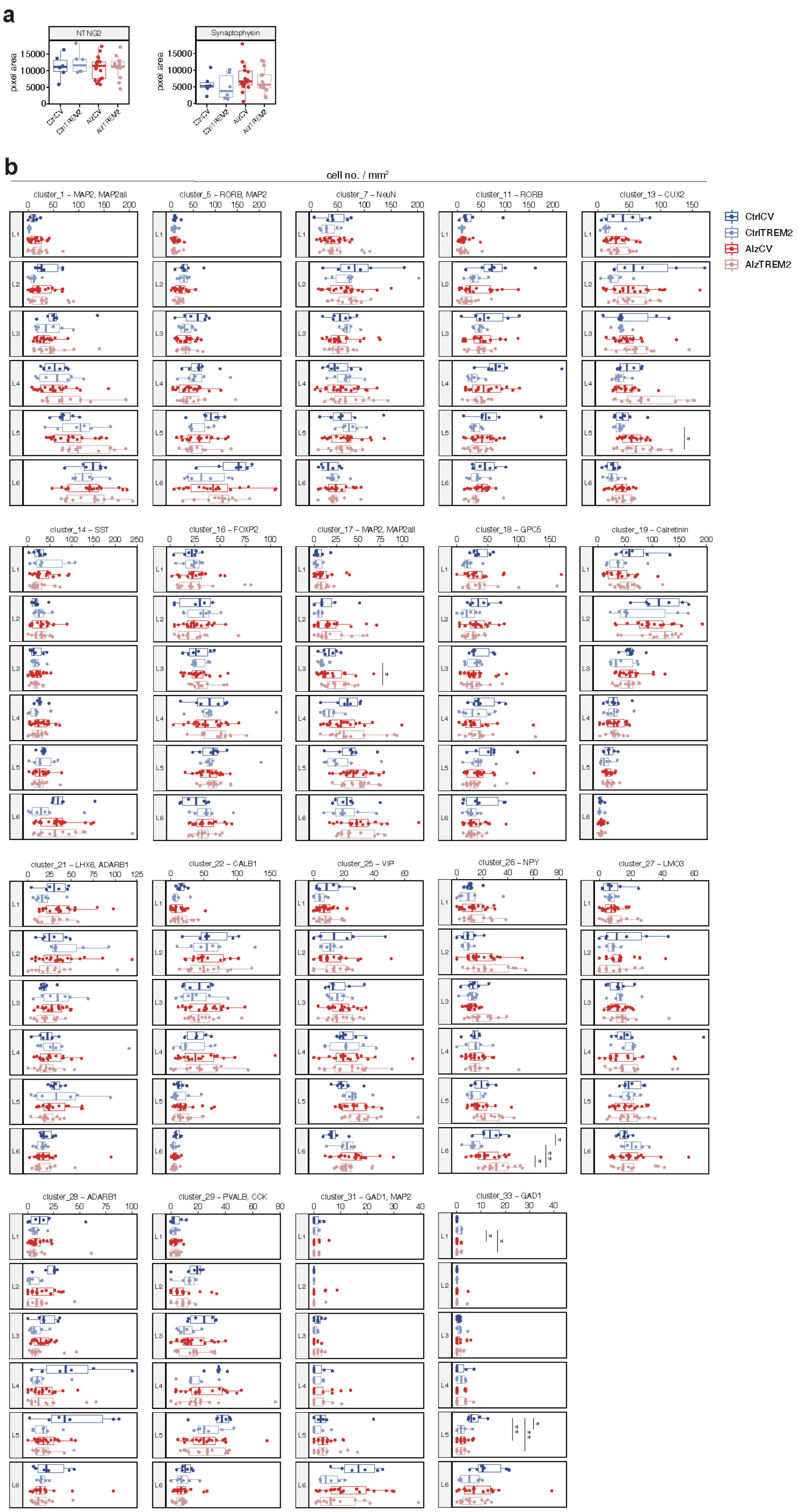
a
NTNG2
Synaptophysin
pixel area
CtrlCV
CtrlTREM2
AlzCV
AlzTREM2
b
cell no. / mm²
cluster_1 – MAP2, MAP2all
cluster_5 – RORB, MAP2
cluster_7 – NeuN
cluster_11 – RORB
cluster_13 – CUX2
cluster_14 – SST
cluster_16 – FOXP2
cluster_17 – MAP2, MAP2all
cluster_18 – GPC5
cluster_19 – Calretinin
cluster_21 – LHX6, ADARB1
cluster_22 – CALB1
cluster_25 – VIP
cluster_26 – NPY
cluster_27 – LMO3
cluster_28 – ADARB1
cluster_29 – PVALB, CCK
cluster_31 – GAD1, MAP2
cluster_33 – GAD1
L1
L2
L3
L4
L5
L6

Supplementary Figure 5. **Synapse marker expression and distributions across cortical layers of all of the neuronal clusters identified. a** Quantification of NTNG2 and synaptophysin signals as marker$^+$ pixel areas in non-diseased control and AD samples carrying *TREM2* common allele and those heterozygotic for *TREM2 R62H or R47H* risk variants (CtrlCV, n=6; CtrlTREM2, n=6; AlzCV, n=18; AlzTREM2, n=13). **b** The densities of nuclei within cortical layers associated with each labelled neuronal cluster are shown. Quantification was performed on three ROIs acquired from a single section of each sample and pooled together before performing statistical analyses between groups. Statistical significance was calculated with Dirichlet regression (a) or either ANOVA and two-sided Tukey tests or Kruskal–Wallis and two-sided Wilcoxon signed-rank test depending on whether groups showed normal or non-normal distributions, respectively (b). Boxplots show median (middle line), interquartile range (box) and variability outside of first and third quartile (lines extending from box). *P* values are indicated as: non-significant, ns, p>0.05; *p≤0.05; **p≤0.01; ***p≤0.001; ****p≤0.0001. Source data are provided as a Source Data file. Original figure can be downloaded from Figshare (https://doi.org/10.6084/m9.figshare.28955909.v1).

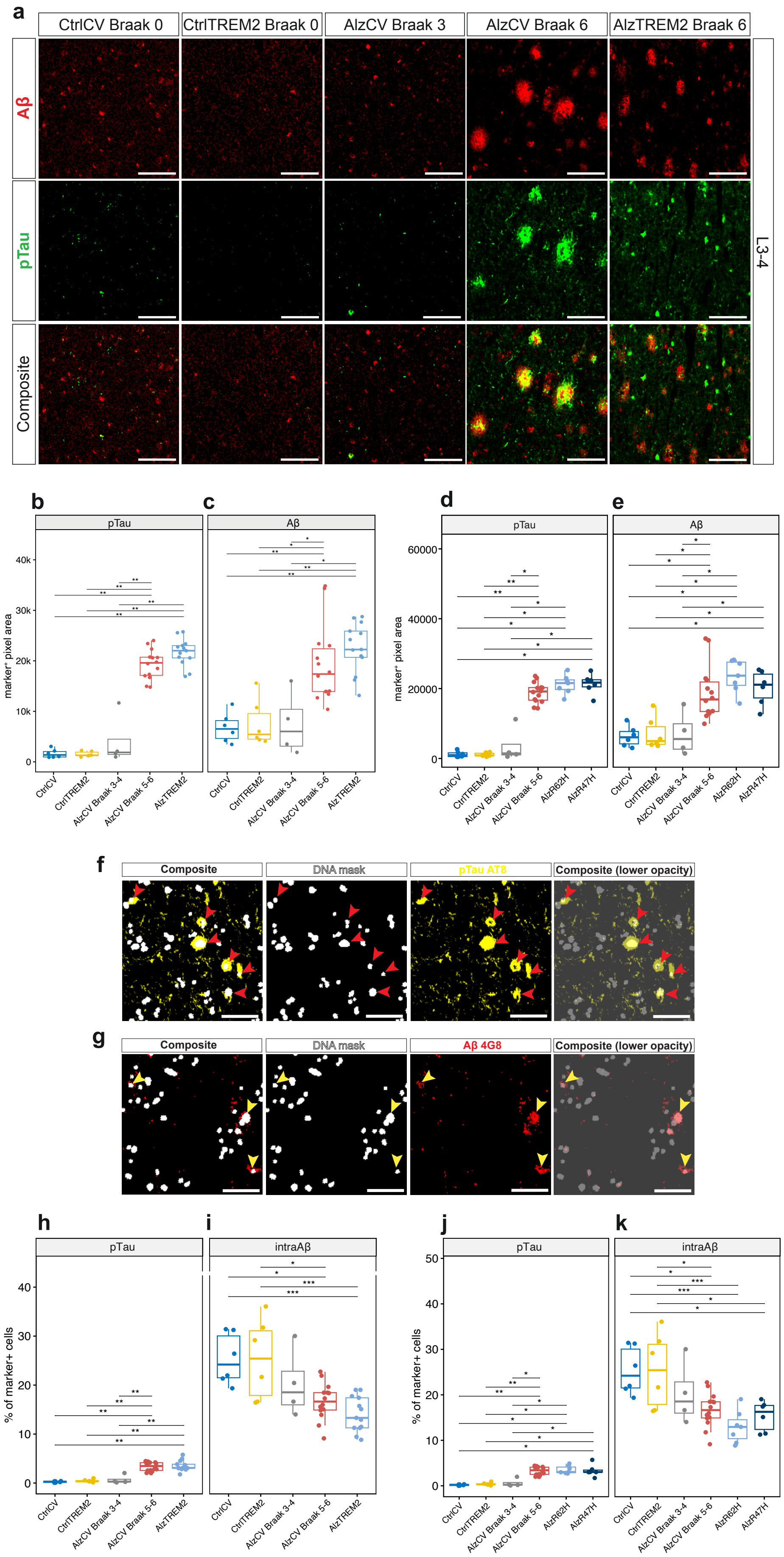
a
CtrlCV Braak 0
CtrlTREM2 Braak 0
AlzCV Braak 3
AlzCV Braak 6
AlzTREM2 Braak 6
Aβ
pTau
Composite
L3-4
b
c
d
e
pTau
Aβ
marker⁺ pixel area
CtrlCV
CtrlTREM2
AlzCV Braak 3-4
AlzCV Braak 5-6
AlzTREM2
AlzR62H
AlzR47H
f
g
Composite
DNA mask
pTau AT8
Composite (lower opacity)
Aβ 4G8
h
i
j
k
intraAβ
% of marker+ cells

Supplementary Figure 6. **Total and intracellular pTau and Aβ accumulation and their correlations with diagnosis or Braak stages. a** Representative IMC images showing total pTau and Aβ signals used for quantifications in (b-e). **b-e** Quantification of total pTau (b,d) and Aβ (c,e) signals as marker^{+} pixel area in non-diseased control and AD samples carrying only the *TREM2* common allele (split by early and late Braak stage) and those heterozygotic for the TREM2 *R62H* or *R47H* risk variants, the latter analysed together (b,c; CtrlCV, n=6; CtrlTREM2, n=6; AlzCV Braak 3-4, n=4; AlzCV Braak 5-6, n=14; AlzTREM2, n=13) or separately (d,e; CtrlCV, n=6; CtrlTREM2, n=6; AlzCV Braak 3-4, n=4; AlzCV Braak 5-6, n=14; AlzR62H, n=7; AlzR47H, n=6). **f,g** Expanded sections of IMC images of the segmented nuclei masks from CtrlTREM2 and AlzTREM2 samples together with pTau AT8 (f) or Aβ 4G8 (g) signals to demonstrate intracellular co-location. pTau^{+} and intraAβ^{+} cells are indicated with red and yellow arrowheads, respectively. **h-k** Proportions of cells positive for pTau (pTau^{+}; h,j) and intracellular Aβ (intraAβ^{+}; I,k) in non-diseased control and AD samples carrying only the *TREM2* common allele (split by early and late Braak stage) or heterozygotic for *R62H* or *R47H* risk variants, the latter analysed together (f,g; CtrlCV, n=6; CtrlTREM2, n=6; AlzCV Braak 3-4, n=4; AlzCV Braak 5-6, n=14; AlzTREM2, n=13) or separately (h,i; CtrlCV, n=6; CtrlTREM2, n=6; AlzCV Braak 3-4, n=4; AlzCV Braak 5-6, n=14; AlzR62H, n=7; AlzR47H, n=6). Quantification was performed on three ROIs acquired from a single section of each sample and pooled together before performing statistical analyses between groups. Statistical significances for comparisons were calculated with either ANOVA and two-sided Tukey tests or Kruskal–Wallis and two-sided Wilcoxon signed-rank test depending on whether groups showed normal or non-normal distributions, respectively (b-e, h-k). Data expressed as percentages (h-k) were transformed with *arcsine* before proceeding with normality and statistical tests. Boxplots show median (middle line), interquartile range (box) and variability outside of first and third quartile (lines extending from box). *P* values are indicated as: non-significant, ns, p>0.05; *p≤0.05; **p≤0.01; ***p≤0.001; ****p≤0.0001. Source data are provided as a Source Data file. Scale bars represent 100μm in (a) and 50μm in (f,g).

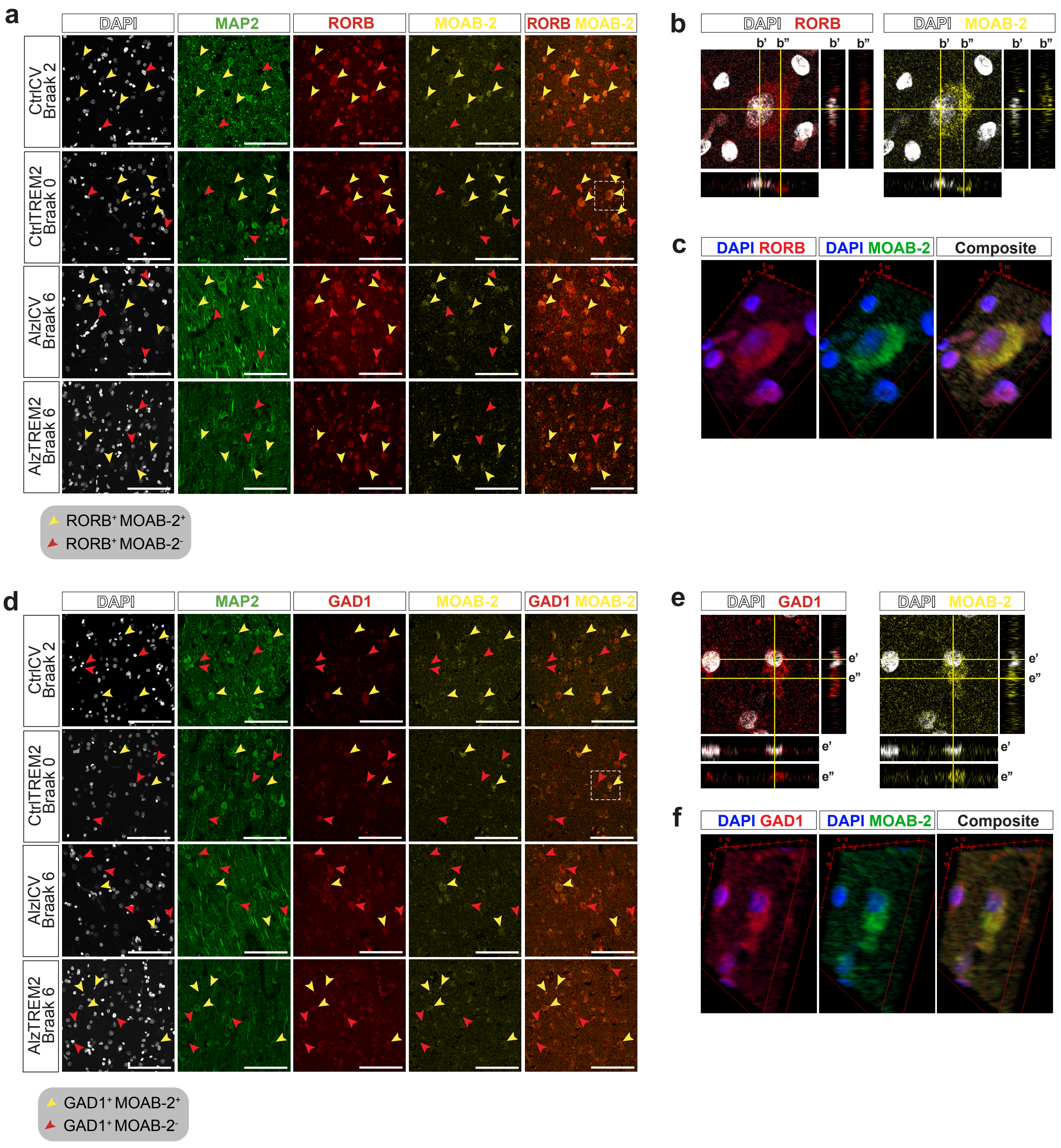

Supplementary Figure 7. **Validation of intraAβ localisation in vulnerable neurons using triple immunofluorescence (IF) microscopy. a,d** Triple IF immunostaining of MTG sections from CtrlCV (Braak 2), CtrlTREM2 (Braak 0), AlzCV (Braak 6) and AlzTREM2 (Braak 6) samples of the indicated groups for the Aβ42-specific antibody MOAB-2, the pan-neuronal marker MAP2 and the vulnerable neuron markers GAD1 (a) and RORB (d). Co-localisation of intraAβ in GAD1^{+} or RORB^{+} neurons is indicated by the yellow arrowheads. **b,c,e,f** Orthogonal projections (b,e) and 3D reconstructions (c,f) of DAPI, MOAB-2 and RORB or GAD1 IF immunostaining of the region delineated by the white dotted square in Ctrl panels to far right of (a) and (d), respectively, showing

co-localisation of MOAB-2/RORB or MOAB-2/GAD1 in the cell body around the nucleus. Scale bars represent 100μm.

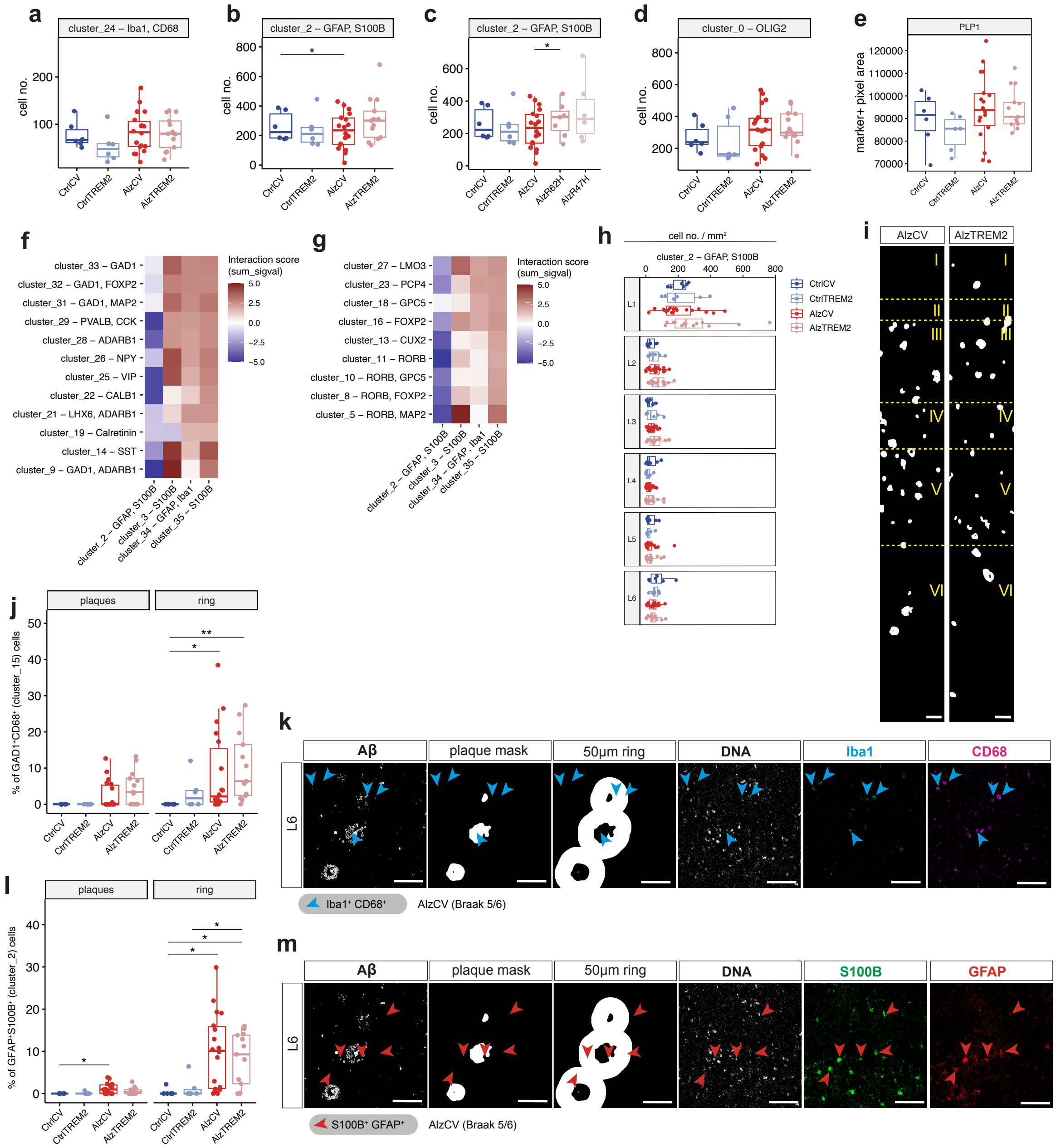

Supplementary Figure 8. **Expression of IMC glial markers and their spatial associations. a,c,d,e** Average number of Iba1⁺CD68⁺ (cluster_24) microglia (a), GFAP⁺S100B⁺ (cluster_2) astrocytes (b,c) and OLIG2⁺ (cluster_0) oligodendrocytes (d) in non-diseased control and AD samples carrying only the *TREM2* common allele or those heterozygotic for *R62H* or *R47H* risk variants, the latter analysed together (a,b,d; CtrlCV, n=6; CtrlTREM2, n=6; AlzCV n=18; AlzTREM2, n=13) or separately (c; CtrlCV, n=6; CtrlTREM2, n=6; AlzCV n=18; AlzR62H, n=7; AlzR47H, n=6). **e** Quantification of PLP1 expression as marker⁺ pixel area in CtrlCV (n=6), CtrlTREM2 (n=6), AlzCV (n=18) and AlzTREM2 (n=13) samples. **f,g** Cell-cell interaction analyses

among astrocyte and inhibitory (f) or excitatory (g) neuronal clusters performed with the SIMPLI "buildSpatialGraph" function. The calculated interaction score "sum_sigval" indicates the rate of interaction (>0) or avoidance (<0) between cell types. **h** Layer-specific changes in densities of GFAP$^+$S100B$^+$ cells (cluster_2) in control and AD samples carrying only the *TREM2* common allele or those heterozygotic for *R62H* or *R47H* risk variants (CtrlCV, n=6; CtrlTREM2, n=6; AlzCV n=18; AlzTREM2, n=13). **g** Representative masks generated from the IMC 4G8$^+$ Aβ channels in AlzCV and AlzTREM2 samples of the types used to identify plaques and for quantification of distributions of these masks across cortical layers as shown in Fig.5.g. **j-m** Quantification of proportions of GAD1$^+$CD68$^+$ microglia (cluster_15; blue arrowheads in k) and GFAP$^+$S100B$^+$ astrocytes (cluster_2; red arrowheads in m) co-localised with plaques and in a 50µm ring around the plaques (j and l, respectively) in CtrlCV (n=6), CtrlTREM2 (n=6), AlzCV (n=18) and AlzTREM2 (n=13) samples. Masks of plaque areas and the surrounding 50µm ring were generated with an ImageJ script (examples of regions obtained are shown in k,m). Quantification was performed on three ROIs acquired from a single section of each sample and pooled together before performing statistical analyses between groups. Statistical significances for comparisons were calculated with Dirichlet regression (a-d) or either ANOVA and two-sided Tukey tests or Kruskal–Wallis and two-sided Wilcoxon signed-rank test depending on whether groups showed normal or non-normal distributions, respectively (e,h,j,l). Boxplots show median (middle line), interquartile range (box) and variability outside of first and third quartile (lines extending from box). *P* values are indicated as: non-significant, ns, p>0.05; *p≤0.05; **p≤0.01; ***p≤0.001; ****p≤0.0001. Source data are provided as a Source Data file. Scale bars in (I,k,m) represent 100μm.

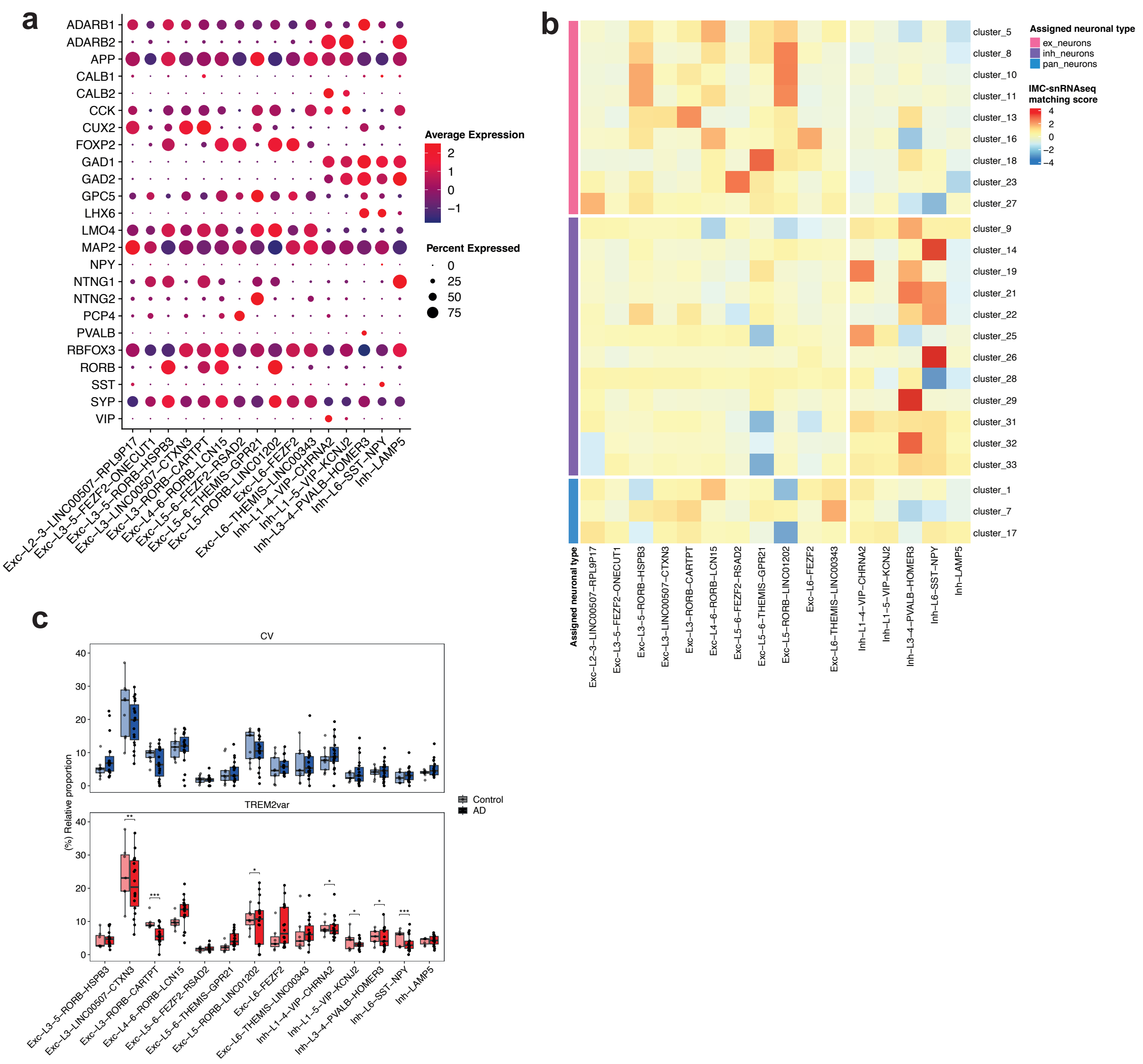

Supplementary Figure 9. **Markers expression and size of snRNAseq clusters and their similarity scores to IMC clusters. a** Average expression (derived by reads number) and percentage of nuclei expressing markers used in imaging mass cytometry (IMC) among snRNAseq neuronal clusters. **b** Summary similarity scores calculated for all IMC-snRNAseq cluster combinations. Coloured boxes on the left highlight groups of neuronal types (excitatory, inhibitory and unclassified neurons) to which IMC clusters were assigned ("Assigned neuronal type"). **c** Changes in relative proportion of size of snRNAseq clusters in AD vs. control samples, either carrying TREM2 common allele (above) or risk variants (below) calculated with Dirichlet regression.

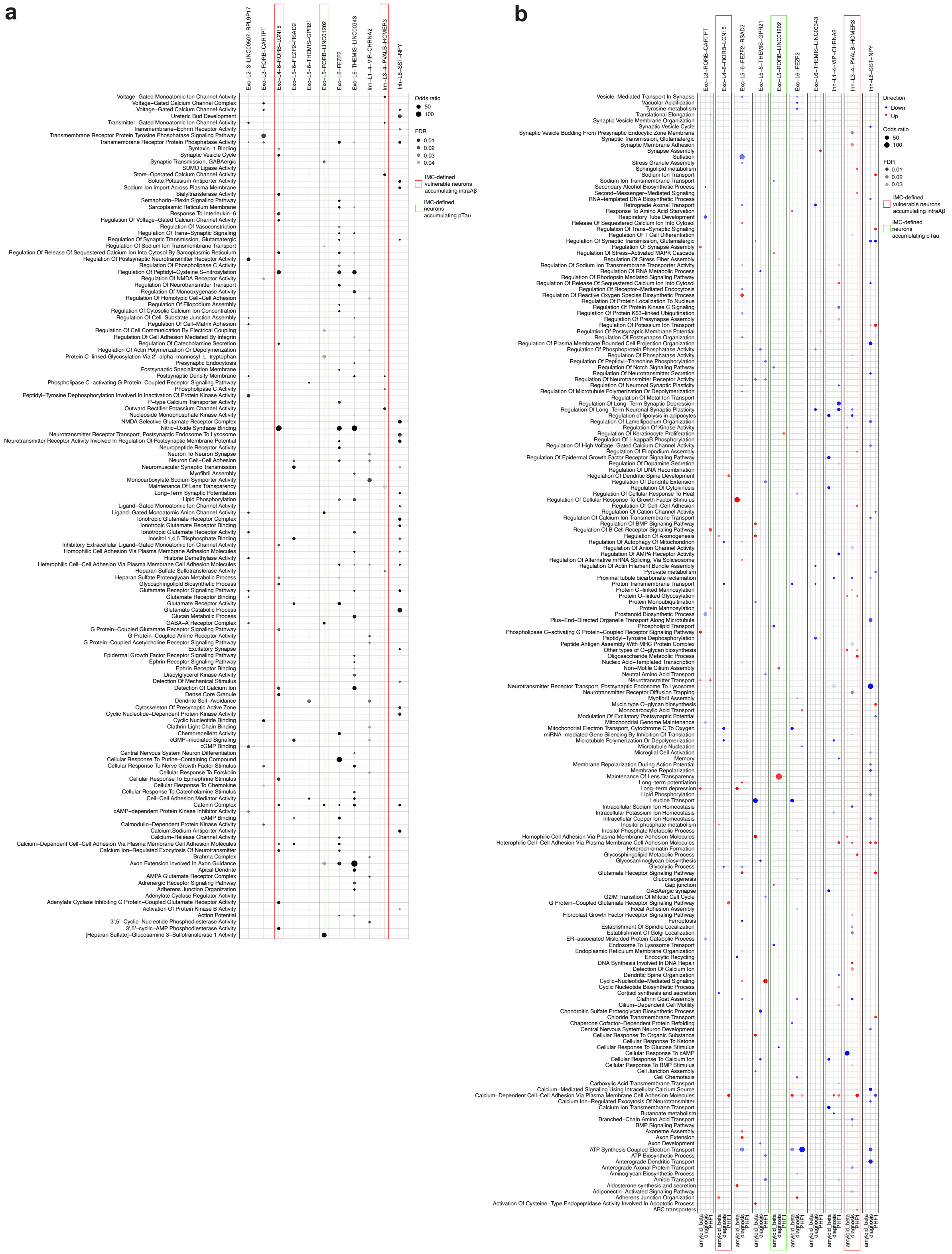

Supplementary Figure 10. **Pathway analysis of transcriptomic changes in snRNAseq clusters and regressed for diagnosis, amyloid and PHF1 expression.**

a Pathway analysis of differentially expressed genes uniquely enriched in each snRNAseq neuronal cluster. Only pathways with FDR ≤ 0.05, odds ratio > 8 and overlapping genes ≥ 3 were analysed. **b** Pathways selectively increased (red) or decreased (blue) in each snRNAseq cluster when regressed for diagnosis, $4G8^+$ amyloid and PHF1 immunostained signal ranked by categories. Imaging mass cytometry (IMC)-defined vulnerable neurons accumulating intraAβ and neurons accumulating pTau are highlighted in red and green, respectively. Only pathways with FDR < 0.04, odds ratio > 8 and overlapping genes ≥ 3 were analysed. Original figure can be downloaded from Figshare (https://doi.org/10.6084/m9.figshare.28955909.v1).

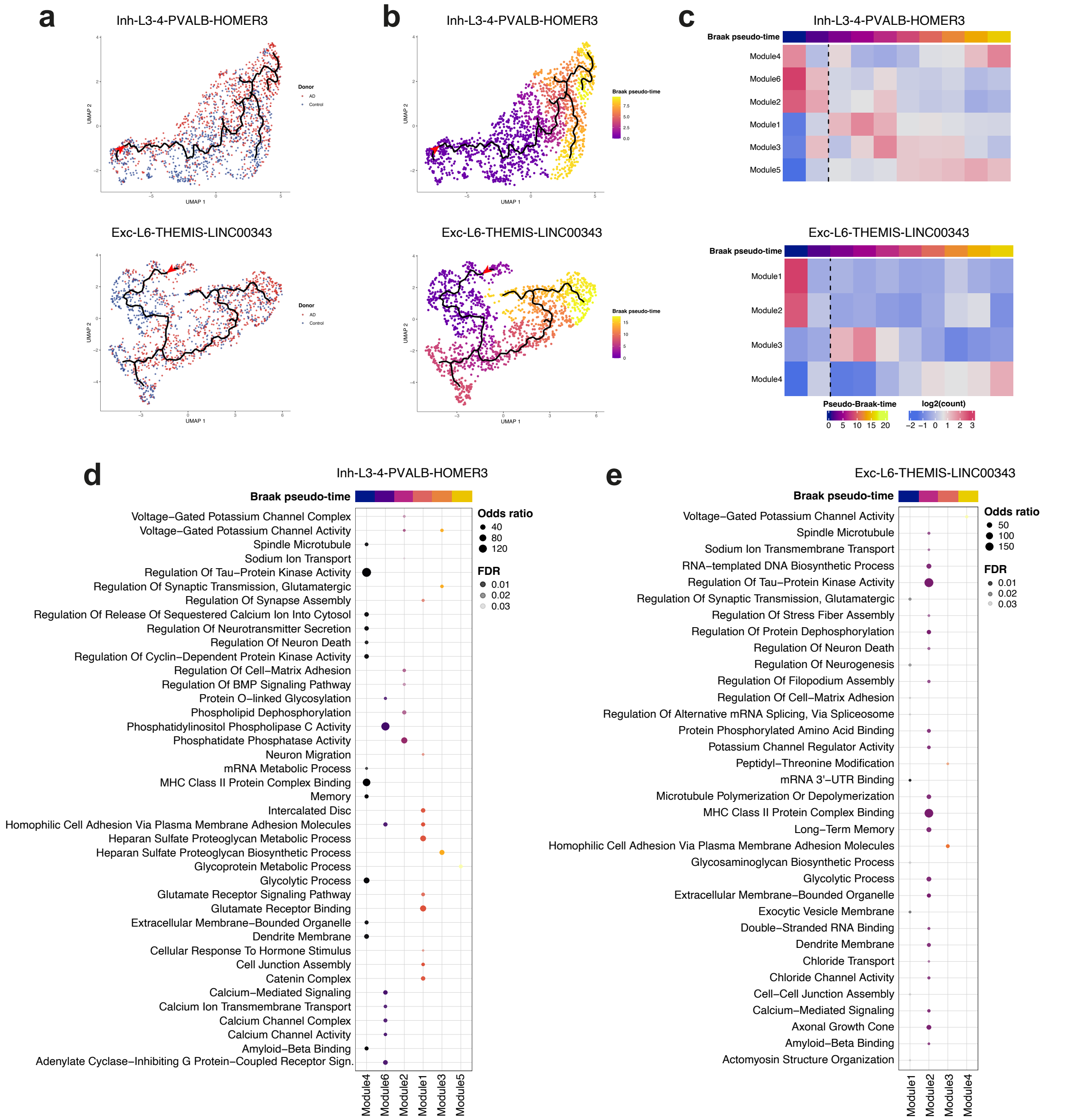

Supplementary Figure 11. **Pathways enriched in Braak pseudo-time trajectory analysis of IMC-snRNAseq matched vulnerable and resilient neuronal clusters. a,b,c** Braak pseudo-time trajectory within clustered nuclei (black line in a,b, trajectory direction indicated by red arrowheads) mimicking disease progression from Braak 0-2 control to Braak 5-6 disease stages and modules of pathways identified enriched at progressive stages of Braak pseudo-time (c) for Inh-L3-4-PVALB-HOMER3 vulnerable (above) and Exc-L6-THEMIS-LINC00343 resilient (below) neuronal clusters. **d,e** Pathways enriched in each module corresponding to consecutive Braak pseudo-time stages identified for Inh-L3-4-PVALB-HOMER3 vulnerable (d) and Exc-L6-THEMIS-

LINC00343 resilient (e) neuronal clusters trajectory analysis. Only pathways with FDR < 0.05, odds ratio > 8 and overlapping genes ≥ 3 were analysed.

Supplementary Tables

Intracellular accumulation of amyloid-ß is a marker of selective neuronal vulnerability in Alzheimer's disease

Alessia Caramello[1,2,3], Nurun Fancy[1], Clotilde Tournerie[1], Maxine Eklund[1], Vicky Chau[1], Emily Adair[1], Marianna Papageorgopoulou[1], Nanet Willumsen[1], Johanna S. Jackson[1], John Hardy[2], Paul M. Matthews[1,4*]

[1] UK Dementia Research Institute Centre at Imperial College London and Department of Brain Sciences, 728 Sir Michael Uren Research Hub, 86 Wood Ln, London W12 0BZ, UK
[2] UK Dementia Research Institute Centre at University College London, Department of Neurodegenerative Disease, Wing 1.2 Cruciform Building, Gower Street, London, WC1E 6BT, UK
[3] Laboratory of Stem Cell Biology and Developmental Genetics, The Francis Crick Institute, 1 Midland Road, London, NW1 1AT, UK
[4] The Rosalind Franklin Institute, Harwell Science and Innovation Campus, Fermi Way, Didcot, Oxon. OX11 0QS, UK

*** Corresponding author:**
Prof Paul M. Matthews
E515, Department of Brain Sciences
Imperial College London
Hammersmith Hospital
DuCane Road, London WC12 0NN
UK
Tel: 0044 207 594 2612
p.matthews@imperial.ac.uk

Supplementary Table 1 - FFPE *post-mortem* samples cohort used for the IMC experiment

Group	CtrlCV	CtrlTREM2	AlzCV	AlzTREM2
**No. of cases**	6	6	18	13
**Mean age (y)**	81.8 ± 8.3	76.3 ± 11.3	76.9 ± 8.5	72.6 ± 11.9
**Age range**	73-92	60-94	64-92	43-89
**Sex (% F)**	33.3%	33.3%	50.0%	53.8%
**Mean PMD (h)**	14.5 ± 6.3	34.8 ± 8.5	24.2 ± 14.1	25.4 ± 11.0
**Mean braak stage**	0.8 ± 1.0	0.8 ± 0.8	5.1 ± 1.1	5.6 ± 0.4
**No. of Braak 5-6 cases**	0	0	14	13
**No. of Braak 3-4 cases**	0	0	4	0
**No. of Braak 0-2 cases**	6	6	0	0
**TREM2 variant (% R47H)**	0.0%	33.3%	0.0%	46.2%

Supplementary Table 2 - Full list of antibodies tested for IMC, with an indication of those selected for final use in IMC (in bold) and/or IF, with corresponding protocol applied

ANTIBODY INFO								IMMUNOSTAINING PROTOCOL			ANTIBODY PURIFICATION		IMC PROTOCOL		
Name	Marker for	Company	Catalog #	Buffer	Host	Clonality	Final use	Dilution	Incubation	Immunostainig result	Antibody type	Kit used	Metal	Dilution	IMC result
**NeuN**	unclassified neurons	Merck	MAB377	0.1% SA	Mouse	mono	IMC	1:500	2x O/N at 4C	Previously optimised in house			Nd148	1:200/1:50/1:30	Good to not great
**MAP2**	unclassified neurons	Abcam	ab236033	PBS	Rabbit	mono	IMC	1:2000	2x O/N at 4C	Previously optimised in house			Gd160	1:1500	Very good
MAP2	unclassified neurons	Abcam	ab302487	59% PBS, 40% Glycerol, 0.05% BSA	Goat	mono	IF	1:100	O/N at 4C	Decent signal					
**MAP2all**	unclassified neurons	LSBio	LS-C163992	ascites in SA 0.09%	Mouse	mono	IMC	1:50/1:250	2x O/N at 4C	Good	Mouse IgG1	Mouse antibody kit	Eu151	1:500/1:200/1:50	Good
MAP2 C/D	unclassified neurons	MyBioSource	MBS500052	PBS + 50% glycerol	Mouse	mono	/	1:2500	2x O/N at 4C	Not great					
LAMPS	excitatory neurons	Invitrogen	14-9778-80	SA 0.09%	Rat	mono	/	1:500	2x O/N at 4C	No specific signal					
**CUX2**	excitatory neurons	Abnova	H00023316-M03	PBS	Mouse	mono	IMC	1:500/1:600	2x O/N at 4C	Good			Nd143	1:200/1:50	Good to not great
GPC5	excitatory neurons	R&D	AF2607-SP	PBS	Goat	poly	/	1:100	O/N at 4C	No specific signal					
**GPC5**	excitatory neurons	abcam	ab248040	PBS	Rabbit	mono	IMC	1:250	O/N at 4C	Very good			Tb159	1:200/1:500/1:50	Good
THEMIS	excitatory neurons	LSBio	LS-C161855	0.09% SA	Rabbit	poly	/	1:100	2x O/N at 4C	No specific signal					
NR4A2	excitatory neurons	LSBio	LS-C99204	0.09% SA	Rabbit	poly	/	1:100	2x O/N at 4C	Good			Yb173	1:50	No specific signal
CDH9	excitatory neurons	LSBio	LS-B8996	0.09% SA	Rabbit	poly	/	1:100	2x O/N at 4C	Some positive cells in upper layers			Dy163	1:200	No specific signal
RORB	excitatory neurons	LSBio	LS-A2564	0.1% SA	Rabbit	poly	IF	1:50	2x O/N at 4C	Decent signal					
RORB	excitatory neurons	abcam	ab188756	0.1% SA in PBS	Rabbit	poly	/	1:200	2x O/N at 4C	Not great			Sm147	1:200/1:50	No specific signal
RORB	excitatory neurons	Sigma	HPA008393	40% glycerol 0.02% SA	Rabbit	mono	/	1:400	1x O/N at 4C	Not great	Rabbit IgG	Protein A kit	Er166	1:200/1:500	No specific signal
**RORB**	excitatory neurons	Sino-biological	202727-T08	PBS, 0.3% proclin	Rabbit	mono	IMC	1:100	1x O/N at 4C	Good			Er166	1:200/1:500	Very good
RPRML	excitatory neurons	LSBio	LS-C817153	0.03% Proclin300	Rabbit	poly	/	1:200	2x O/N at 4C	No specific signal					
COL5A2	excitatory neurons	LSBio	LS-B15862	15mM SA	Rabbit	poly	/	1:200	2x O/N at 4C	Few postive cells					
TOX	excitatory neurons	ThermoFisher	PA5-34423	0.02% SA	Rabbit	poly	/	1:50	2x O/N at 4C	Few postive cells			Nd145	1:200/1:50	No specific signal
PRSS12	excitatory neurons	ThermoFisher	PA5-20363	0.02% SA	Rabbit	poly	/	1:400/1:200	2x O/N at 4C	Few postive cells			Pr141	1:200/1:50	No specific signal
TLE4	excitatory neurons	LSBio	LS-C137217	0.02% SA	Rabbit	poly	/	1:200	2x O/N at 4C	No specific signal					
TLE4	excitatory neurons	Santa Cruz	sc-365406	PBS, 0.1 SA, 0.1% gelatin	Mouse	mono	/	1:200	1x O/N at 4C	No specific signal					
ROBO3	excitatory neurons	LSBio	LS-C817143	0.03% Proclin300	Rabbit	poly	/	1:200	2x O/N at 4C	No specific signal					
ROBO3	excitatory neurons	Sino-biological	206770-T08	PBS, pH7.0 with 0.03% Proclin300	Rabbit	poly	/	1:100	1x O/N at 4C	No specific signal					
**PCP4**	excitatory neurons	Sino-biological	207078-T08	PBS, pH7.0 with 0.03% Proclin300	Rabbit	poly	IMC	1:200	1x O/N at 4C	Very good			Gd155	1:50/1:200/1:500	Few postive cells
**FOXP2**	excitatory neurons	Sino-biological	204066-T08	PBS, pH7.0 with 0.03% Proclin300	Rabbit	poly	IMC	1:200	1x O/N at 4C	Not great			Dy161	1:50/1:200/1:500	Few postive cells
**LMO4**	excitatory neurons	Sino-biological	201969-T08	PBS, pH7.0 with 0.03% Proclin300	Rabbit	poly	IMC	1:200	1x O/N at 4C	Not great			Sm149	1:50/1:200/1:500	Few postive cells
CALB1	inhibitory neurons	Swant	300pur	PBS	Mouse	mono	/	1:500/1:2000	2x O/N at 4C	Good	Mouse IgG1	Protein G kit	Sm152	1:200/1:50	No specific signal
**CALB1**	inhibitory neurons	Abcam	ab233018	PBS	Rabbit	mono	IMC	1:500	2x O/N at 4C	Good			Sm152	1:500/1:200/1:50	Very good
CALB1	inhibitory neurons	Sino-biological	102051-T46	0.2 µm filtered solution in PBS	Rabbit	poly	/	1:1000	1x O/N at 4C	Few weakly postive cells					
VIP	inhibitory neurons	NovusBio	NBP1-05163-0.025ml	15mM SA	Mouse	mono	/	1:200/1:100	2x O/N at 4C	No specific signal					
VIP	inhibitory neurons	Abcam	ab22736	0.1% SA + serum	Rabbit	poly	/	1:500	2x O/N at 4C	Few weakly postive cells corresponding to MAP2 signal					
VIP	inhibitory neurons	Abcam	ab272726	0.01% SA + 40% glycerol + 0.05% BSA	Rabbit	mono	/	1:500	2x O/N at 4C	Few postive cells	Rabbit IgG	Protein G kit	Eu153	1:500/1:200/1:50/1:20	No specific signal
**VIP**	inhibitory neurons	Abcam	ab273589	PBS	Rabbit	mono	IMC			(carrier-free version of ab272726)			Gd158	1:200/1:500/1:50	Good to not great
RELN	inhibitory neurons	NovusBio	NB600-1080	0.1% SA	Mouse	mono	/	1:100/1:200	2x O/N at 4C	Some positive cells in upper layers			Yb171	1:200/1:50	Few weakly positive cells
RELN	inhibitory neurons	Abcam	ab78540	0.1% SA, 1.45% NaCl, 0.242% Tris	Mouse	mono	/		2x O/N at 4C	No specific signal					
GAD1	inhibitory neurons	NovusBio	NBP3-08276	PBS	Mouse	mono	/	1:1000	2x O/N at 4C	Very good			Nd143	1:200/1:50	Not great
GAD1	inhibitory neurons	MyBioSource	MBS4380442	PBS	Mouse	mono	/	1:100	2x O/N at 4C	Not great			Nd143	1:200/1:50/1:20	Good to not great
GAD1	inhibitory neurons	MyBioSource	MBS4380441	PBS	Mouse	mono	/	1:1000	2x O/N at 4C	No specific signal					
GAD1	inhibitory neurons	Abcam	ab246335	PBS	Rabbit	mono	/	1:500	2x O/N at 4C	Very good			Nd143	1:500/1:200/1:50	Not great
**GAD65/67**	inhibitory neurons	Abcam	ab240280	PBS	Rabbit	mono	IMC/IF	1:200	2x O/N at 4C	Very good			Yb172	1:200/1:500	Good
**NPY**	inhibitory neurons	LSBio	LS-B6400	PBS	Mouse	mono	IMC	1:100	2x O/N at 4C	Very good			Er170	1:200/1:50	Very good
**LHX6**	inhibitory neurons	LSBio	LS-B10690	0.09% SA	Rabbit	poly	IMC	1:75	2x O/N at 4C	Very good			Yb176	1:200/1:50	Good
NDNF	inhibitory neurons	LSBio	LS-C168140	0.09% SA	Rabbit	poly	/	1:75	2x O/N at 4C	Some positive cells in upper layers			Er167	1:50	No specific signal
**CCK**	inhibitory neurons	LSBio	LS-C177585	0.05% SA	Rabbit	poly	IMC	1:100	2x O/N at 4C	Few weakly postive cells			Nd142	1:200/1:50	Good to not great
CCK	inhibitory neurons	Abcam	ab27441	PBS, 1% BSA	Rabbit	poly	/	1:200	2x O/N at 4C	Few weakly postive cells	Rabbit IgG	Protein A kit	Nd142	1:500/1:200/1:50	No specific signal
**ADARB1**	inhibitory neurons	LSBio	LS-C176789	15mM SA	Rabbit	poly	IMC	1:200	2x O/N at 4C	Very good			Lu175	1:500/1:200/1:50	Good to not great
**Calretinin**	inhibitory neurons	Abcam	ab232462	PBS	Rabbit	mono	IMC	1:250	O/N at 4C	Good			Nd145	1:500/1:200/1:50	Very good
**PVALB**	inhibitory neurons	NovusBio	NB120-11427	PBS, 0.02% SA	Rabbit	poly	IMC			Previously optimised in house			Dy163	1:1000	Very good
**SST**	inhibitory neurons	Abcam	ab108456	PBS, 0.02% SA	Rabbit	poly	IMC			Previously optimised in house			Er168	1:200/1:500	Good to not great
**NTNG2**	synapses	LSBio	LS-B4540	PBS	Mouse	mono	IMC	1:200	2x O/N at 4C	Good			Nd150	1:500/1:200/1:50	Good to not great
**Synoptophysin**	synapses	Abcam	ab214621	PBS	Rabbit	mono	IMC			Previously optimised in house			Pr141	1:200/1:500	Weak signal
**OLIG2**	oligodendrocytes	Abcam	ab220796	PBS	Rabbit	mono	IMC			Previously optimised in house			Gd156	1:100/1:200/1:500	Good to not great
**PLP1**	myelin	Invitrogen	MA1-80034	0.1% SA	Mouse	mono	IMC			Previously optimised in house					Already conjugated in house
**S100B**	astrocytes	NovusBio	NBP2-53188	PBS with 0.05% BSA, 0.05% SA	Rabbit	mono	IMC			Previously optimised in house					Already conjugated in house
**GFAP**	astrocytes	Abcam	ab218309	PBS	Rabbit	mono	IMC			Previously optimised in house			Dy162	1:500	Very good
**Iba1**	microglia	WAKO	019-197471	TBS	Rabbit	poly	IMC			Previously optimised in house					Already conjugated in house
Iba1	microglia	Abcam	ab5076	0.02% SA, Tris buffered saline, 0.5% BSA	Goat	poly	IF	1:500	O/N at 4C	Good					
**CD68**	microglia	Abcam	ab227458	PBS	Rabbit	mono	IMC			Previously optimised in house			Yb174	1:500/1:100	Good
CD68	microglia	Abcam	ab955	0.01% SA, PBS, 40% glycerol, 0.05% BSA	Mouse	mono	IF	1:200	O/N at 4C	Good					
**APP**	AD pathology	Thermofisher	13-0200	PBS, 0.1% SA	Mouse	mono	IMC			Previously optimised in house					Already conjugated in house
**Amyloids (4G8)**	AD pathology	BioLegend	800702	PBS	Mouse	mono	IMC			Previously optimised in house			Nd144	1:500	Very good
Ab42 (MOAB-2)	AD pathology	NovusBio	NBP2-13075	PBS, 0.05% SA	Mouse	mono	IF	1:100	O/N at 4C	Decent signal					
**pTau (AT8)**	AD pathology	Thermofisher	MN1020	PBS	Mouse	mono	IMC			Previously optimised in house			Ho165	1:200/1:500	Good

Supplementary Table 3 - Final IMC antibody panel used with corresponding conjugated metal and concentration applied

Metal conjugate	Antibody	Catalogue #	Dilution
141 Pr	Synaptophysin	ab214621	1:200
142 Nd	CCK	LS-C177585	1:200
143 Nd	CUX2	H00023316-M03	1:60
144 Nd	Ab	800702	1:500
145 Nd	Calretinin	ab232462	1:500
148 Nd	NeuN	MAB377	1:30
149 Sm	LMO3	201969-T08	1:50
150 Nd	NTNG2	LS-B4540	1:500
151 Eu	MAP2 all	LS-C163992	1:500
152 Sm	CALB1	ab233018	1:300
154 Sm	APP	13-0200	1:400
155 Gd	PCP4	207078-T08	1:50
156 Gd	OLIG2	ab220796	1:100
158 Gd	VIP	ab273589	1:50
159 Tb	GPC5	ab248040	1:50
160 Gd	MAP2	ab236033	1:1500
161 Dy	FOXP2	204066-T08	1:50
162 Dy	GFAP	ab218309	1:500
163 Dy	PVALB	NB120-11427	1:1000
165 Ho	pTau	MN1020	1:200
166 Er	RORB	202727-T08	1:200
167 Er	S100B	NBP2-53188	1:1000
168 Er	SST	ab108456	1:200
169 Tm	Iba1	019-197471	1:3000
170 Er	NPY	LS-B6400	1:500
171 Yb	PLP1	MA1-80034	1:1000
172 Yb	GAD1	ab240280	1:200
174 Yb	CD68	ab227458	1:90
175 Lu	ADARB1	LS-C176789	1:50
176 Yb	LHX6	LS-B10690	1:50
191/193Ir	/	201192A	1:400

Supplementary Table 4 - Fresh frozen *post-mortem* samples cohort for the snRNAseq experiment

Group	CtrlCV	CtrlTREM2	AlzCV	AlzTREM2
**No. of cases**	9	7	19	18
**Mean age (y)**	81 ± 8.9	74.7 ± 11.2	77 ± 8.2	71.1 ± 11.0
**Age range**	67-92	60-94	64-92	43-89
**Sex (% F)**	44.4%	28.6%	52.6%	50.0%
**Mean PMD (h)**	16.3 ± 6.3	33 ± 9.2	25 ± 14.2	28.7 ± 11.9
**Mean braak stage**	0.9 ± 0.9	0.7 ± 0.7	5.1 ± 1.1	5.6 ± 0.4
**No. of Braak 5-6 cases**	0	0	15	18
**No. of Braak 3-4 cases**	0	0	4	0
**No. of Braak 0-2 cases**	9	7	0	0
**TREM2 variant (% R47H)**	0.0%	28.6%	0.0%	44.4%

Supplementary Table 5 - Final IMC-snRNAseq clusters match

IMC cluster	snRNAseq cluster	common markers	IMC cluster label	summary similarity score
cluster_1	Exc-L4-6-RORB-LCN15	FOXP2, LMO4, RORB, MAP2	MAP2, MAP2all	59.01
cluster_5	Exc-L4-6-RORB-LCN15	LMO4, RORB, MAP2	RORB, MAP2	34.98
cluster_7	Exc-L6-THEMIS-LINC00343	MAP2, LMO4, ADARB1	NeuN	12.93
cluster_8	Exc-L4-6-RORB-LCN15	LMO4, MAP2, RORB, FOXP2	RORB, FOXP2	50.95
cluster_9	Inh-L3-4-PVALB-HOMER3	GPC5, GAD1	GAD1, ADARB1	32.45
cluster_10	Exc-L5-RORB-LINC01202	GPC5, ADARB1, CUX2, LMO4, RORB	RORB, GPC5	370.20
cluster_11	Exc-L5-RORB-LINC01202	FOXP2, LMO4, ADARB1, RORB	RORB	102.13
cluster_13	Exc-L3-RORB-CARTPT	LMO4, RORB, ADARB1, CUX2	CUX2	200.29
cluster_14	Inh-L6-SST-NPY	LHX6, SST	SST	207.64
cluster_16	Exc-L6-FEZF2	GPC5, LMO4, FOXP2	FOXP2	97.26
cluster_17	Exc-L2-3-LINC00507-RPL9P17	ADARB1, LMO4, MAP2	MAP2, MAP2all	10.24
cluster_18	Exc-L5-6-THEMIS-GPR21	ADARB1, NTNG2, LMO4, GPC5	GPC5	122.23
cluster_19	Inh-L1-4-VIP-CHRNA2	VIP, ADARB1, Calretinin	Calretinin	399.30
cluster_21	Inh-L3-4-PVALB-HOMER3	ADARB1, LHX6	LHX6, ADARB1	118.92
cluster_22	Inh-L6-SST-NPY	LHX6, CALB1	CALB1	203.63
cluster_23	Exc-L5-6-FEZF2-RSAD2	ADARB1, LMO4, MAP2, PCP4	PCP4	151.61
cluster_25	Inh-L1-4-VIP-CHRNA2	ADARB1, MAP2, VIP	VIP	60.38
cluster_26	Inh-L6-SST-NPY	GPC5, LHX6, NPY	NPY	111.17
cluster_27	Exc-L2-3-LINC00507-RPL9P17	MAP2, ADARB1, LMO4	LMO3	38.28
cluster_28	Inh-L3-4-PVALB-HOMER3	GAD1, LHX6, ADARB1	ADARB1	8.90
cluster_29	Inh-L3-4-PVALB-HOMER3	GAD1, ADARB1, PVALB	PVALB, CCK	255.93
cluster_31	Inh-L1-4-VIP-CHRNA2	VIP, MAP2, GAD1	GAD1, MAP2	23.12
cluster_32	Inh-L3-4-PVALB-HOMER3	ADARB1, GAD1	GAD1, FOXP2	78.11
cluster_33	Inh-L3-4-PVALB-HOMER3	CUX2, GAD1	GAD1	19.06
