# Supplementary material for: Intracellular accumulation of amyloid-ß is a marker of selective neuronal vulnerability in Alzheimer’s disease
Source: Nat Commun. 2025 Jun 4;16:5189. doi: 10.1038/s41467-025-60328-w (PMC12137956; doi:10.1038/s41467-025-60328-w)
Supplement: Supplementary file 2 — Description of Additional Supplementary Files [file 41467_2025_60328_MOESM2_ESM.docx]

**Description of Additional Supplementary Files**

**File name:** Supplementary Data 1-4
**Description:**

Supplementary Data 1: Similarity scores calculated for all IMC-snRNAseq clusters combination to identify the best IMC-RNAseq matching clusters.

Supplementary Data 2: Pathways enriched in each snRNAseq neuronal cluster contrasted to the others.

Supplementary Data 3: Pathways enriched in each snRNAseq neuronal cluster regressed for diagnosis, PHF1 or b-amyloid immunostaining.

Supplementary Data 4: Pathways enriched in trajectory analysis of vulnerable and resilient neurons.
