# Supplementary material for: Intracellular accumulation of amyloid-ß is a marker of selective neuronal vulnerability in Alzheimer’s disease
Source: Nat Commun. 2025 Jun 4;16:5189. doi: 10.1038/s41467-025-60328-w (PMC12137956; doi:10.1038/s41467-025-60328-w)
Supplement: Supplementary file 4 — Reporting Summary [file 41467_2025_60328_MOESM4_ESM.pdf]

nature portfolio

Corresponding author(s): Paul M Matthews

Last updated by author(s): 04/04/2025

Reporting Summary

Nature Portfolio wishes to improve the reproducibility of the work that we publish. This form provides structure for consistency and transparency in reporting. For further information on Nature Portfolio policies, see our Editorial Policies and the Editorial Policy Checklist.

Please do not complete any field with "not applicable" or n/a. Refer to the help text for what text to use if an item is not relevant to your study.
For final submission: please carefully check your responses for accuracy; you will not be able to make changes later.

Statistics

For all statistical analyses, confirm that the following items are present in the figure legend, table legend, main text, or Methods section.

n/a	Confirmed	
☐	☒	The exact sample size (*n*) for each experimental group/condition, given as a discrete number and unit of measurement
☐	☒	A statement on whether measurements were taken from distinct samples or whether the same sample was measured repeatedly
☐	☒	The statistical test(s) used AND whether they are one- or two-sided<br>*Only common tests should be described solely by name; describe more complex techniques in the Methods section.*
☒	☐	A description of all covariates tested
☐	☒	A description of any assumptions or corrections, such as tests of normality and adjustment for multiple comparisons
☒	☐	A full description of the statistical parameters including central tendency (e.g. means) or other basic estimates (e.g. regression coefficient) AND variation (e.g. standard deviation) or associated estimates of uncertainty (e.g. confidence intervals)
☐	☒	For null hypothesis testing, the test statistic (e.g. *F*, *t*, *r*) with confidence intervals, effect sizes, degrees of freedom and *P* value noted<br>*Give P values as exact values whenever suitable.*
☒	☐	For Bayesian analysis, information on the choice of priors and Markov chain Monte Carlo settings
☒	☐	For hierarchical and complex designs, identification of the appropriate level for tests and full reporting of outcomes
☒	☐	Estimates of effect sizes (e.g. Cohen's *d*, Pearson's *r*), indicating how they were calculated

Our web collection on statistics for biologists contains articles on many of the points above.

Software and code

Policy information about availability of computer code

Data collection	No code was used for collecting data.
Data analysis	SIMPLI (Bortolomeazzi, M. et al, 2022) was used for analysing the IMC images, Rstudio (version 2023.06.2+561) was used for data analysis and plotting, scFlow (Khozoie, C. et al, 2021) was used for snRNAseq dataset processing and pathway analysis. R scripts used for data analysis are available on a public repository (https://github.com/AlessiaCaramello/Vulnerable-neurons-in-AD). Immunofluorescence and IMC images were processed with ImageJ (version 2.14.0/1.54f). Figures were assembled with Adobe Illustrator (version 28.0). The 2D k-nearest neighbours of nuclei included in the neuronal and glial clusters were detected using the buildSpatialGraph function included in the imcRtools R/Bioconductor package (version 1.0.2). Impacted pathway analysis (IPA) was performed essentially as previously described using the enrichR (v 3.3) package.

For manuscripts utilizing custom algorithms or software that are central to the research but not yet described in published literature, software must be made available to editors and reviewers. We strongly encourage code deposition in a community repository (e.g. GitHub). See the Nature Portfolio guidelines for submitting code & software for further information.

Data

Policy information about availability of data

All manuscripts must include a data availability statement. This statement should provide the following information, where applicable:

- Accession codes, unique identifiers, or web links for publicly available datasets
- A description of any restrictions on data availability
- For clinical datasets or third party data, please ensure that the statement adheres to our policy

Source Data for all plots are provided with this paper. All raw IMC images and their normalised and preprocessed version generated with SIMPLI, as well as images generated upon nuclei channel segmentation in SIMPLI as available to download from figshare (10.6084/m9.figshare.27909663). The two original datasets generated by SIMPLI (area_measurements.csv and clustered_cells.csv) containing all the IMC data showed in this study are also available to download from figshare (10.6084/m9.figshare.27901113). The snRNAseq dataset analysed in this study (MTG samples only) is available to download from Synapse (Project ID: syn36812517).

Research involving human participants, their data, or biological material

Policy information about studies with human participants or human data. See also policy information about sex, gender (identity/presentation), and sexual orientation and race, ethnicity and racism.

Reporting on sex and gender	Sex of human samples used has been disclosed, based on information collected by the UK brain bank from where samples were requested. Sex of the tissue samples is reported in Supp Table 1.
Reporting on race, ethnicity, or other socially relevant groupings	This information was not available for the samples used in this study.
Population characteristics	Diagnostic information (healthy or diagnosed with AD, Braak stage, expression of TREM2 variant) were used for grouping samples in this study, based on information collected by the UK brain bank from where samples were requested.
Recruitment	Samples were selected exclusively on diagnostic information.
Ethics oversight	This study was carried out in accordance with the Regional Ethics Committee and Imperial College Use of Human Tissue guidelines.

Note that full information on the approval of the study protocol must also be provided in the manuscript.

Field-specific reporting

Please select the one below that is the best fit for your research. If you are not sure, read the appropriate sections before making your selection.

☒ Life sciences ☐ Behavioural & social sciences ☐ Ecological, evolutionary & environmental sciences

For a reference copy of the document with all sections, see nature.com/documents/nr-reporting-summary-flat.pdf

Life sciences study design

All studies must disclose on these points even when the disclosure is negative.

Sample size	Samples used in this study were already used previously in the group for a previous study (Fancy et al., 2022), therefore sample size was not changed.
Data exclusions	IMC images quality was scored and only images meeting the minimum quality was processed for data analysis. No data was excluded.
Replication	Accuracy and precision of the IMC antibody panel in detecting neuronal populations was tested by the possibility of identifying the same neuronal populations in an independent IMC staining (3 consecutive slides from CtrlTREM2 tissue) and determining the coefficient of variation among slides and among patients (see methods section "Evaluation of accuracy and precision of IMC antibody panel").
Randomization	Samples were grouped based on diagnostic information.
Blinding	Data was automatically processed with SIMPLI and Rstudio and therefore blinding was not necessary.

Reporting for specific materials, systems and methods

We require information from authors about some types of materials, experimental systems and methods used in many studies. Here, indicate whether each material, system or method listed is relevant to your study. If you are not sure if a list item applies to your research, read the appropriate section before selecting a response.

Materials & experimental systems

n/a	Involved in the study
☐	☒ Antibodies
☒	☐ Eukaryotic cell lines
☒	☐ Palaeontology and archaeology
☒	☐ Animals and other organisms
☒	☐ Clinical data
☒	☐ Dual use research of concern
☒	☐ Plants

Methods

n/a	Involved in the study
☒	☐ ChIP-seq
☒	☐ Flow cytometry
☒	☐ MRI-based neuroimaging

Antibodies

Antibodies used

Synaptophysin (ab214621), CCK (LS-C177585), CUX2 (H00023316-M03), Ab 4G8 (800702), Ab MOAB-2 (NBP2-13075), Calretinin (ab232462), NeuN (MAB377), LMO3 (201969-T08), NTNG2 (LS-B4540), MAP2 all (LS-C163992), CALB1 (ab233018), APP (13-0200), PCP4 (207078-T08), OLIG2 (ab220796), VIP (ab273589), GPC5 (ab248040), MAP2 (ab236033, ab302487), FOXP2 (204066-T08), GFAP (ab218309), PVALB (NB120-11427), pTau (MN1020), RORB (202727-T08), S100B (NBP2-53188), SST (ab108456), Iba1 (019-197471, ab5076), NPY (LS-B6400), PLP1 (MA1-80034), GAD1 (ab240280), CD68 (ab227458, ab955), ADARB1 (LS-C176789), LHX6 (LS-B10690)

Validation

All new antibodies that were not previously been tested in the lab, were validated with immunofluorescence on FFPE control human prefrontal cortex tissue. Staining signal was compared to what available in the literature and on manufacturer websites. Neuronal specificity of neuronal markers was checked by co-staining with MAP2. See also "Designing and testing of the neuronal antibody panel for IMC" section in Methods.

Plants

Seed stocks

Report on the source of all seed stocks or other plant material used. If applicable, state the seed stock centre and catalogue number. If plant specimens were collected from the field, describe the collection location, date and sampling procedures.

Novel plant genotypes

Describe the methods by which all novel plant genotypes were produced. This includes those generated by transgenic approaches, gene editing, chemical/radiation-based mutagenesis and hybridization. For transgenic lines, describe the transformation method, the number of independent lines analyzed and the generation upon which experiments were performed. For gene-edited lines, describe the editor used, the endogenous sequence targeted for editing, the targeting guide RNA sequence (if applicable) and how the editor was applied.

Authentication

Describe any authentication procedures for each seed stock used or novel genotype generated. Describe any experiments used to assess the effect of a mutation and, where applicable, how potential secondary effects (e.g. second site T-DNA insertions, mosiacism, off-target gene editing) were examined.

This checklist template is licensed under a Creative Commons Attribution 4.0 International License, which permits use, sharing, adaptation, distribution and reproduction in any medium or format, as long as you give appropriate credit to the original author(s) and the source, provide a link to the Creative Commons license, and indicate if changes were made. The images or other third party material in this article are included in the article's Creative Commons license, unless indicated otherwise in a credit line to the material. If material is not included in the article's Creative Commons license and your intended use is not permitted by statutory regulation or exceeds the permitted use, you will need to obtain permission directly from the copyright holder. To view a copy of this license, visit http://creativecommons.org/licenses/by/4.0/
